# Non-binary experiences of (gender-based) violence at work

**DOI:** 10.1080/09585192.2025.2507964

**Published:** 2025-05-21

**Authors:** Anne Laure Humbert, Charikleia (Charoula) Tzanakou, Sofia Strid, Anke Lipinsky

**Affiliations:** a Department of Sociology and Work Science, University of Gothenburg, Gothenburg, Sweden; b Centre for Diversity Policy Research and Practice, Oxford Brookes University, Oxford, UK; c Center of Excellence Women and Science, GESIS - Leibniz Institute for the Social Sciences, Mannheim, Germany

**Keywords:** Non-binary, LGBT, gender-based violence, psychological violence, sexual harassment, diversity management

## Abstract

The experiences of non-binary people remain underexplored in HRM research. With limited knowledge and evidence, putting in place diversity management policies and practices is challenging. This article advances understandings of the experiences of non-binary people at work by providing empirical evidence from a survey conducted in 15 countries across Europe with nearly 18,000 staff at universities and research organisations, including 173 who identified as non-binary. Results suggest that non-binary people are more likely than other gender identity groups to feel socially excluded and unsafe at work, as well as more likely to be subjected to psychological violence and sexual harassment. The analysis shows that experiences of gender-based violence mediate the relationship between being non-binary and feeling socially excluded, unsafe or unwell at work. By integrating minority stress theory into the study of workplace inequalities, this research deepens the understanding of how systemic stigmatisation operates within gendered and binarist organisations. These findings emphasise the need to integrate considerations of gender-based violence into diversity management interventions and disrupt binary gender norms to ensure inclusion and safety at work. By advancing diversity, equality, and inclusion scholarship, this article provides actionable insights for HRM practitioners to address the unique challenges faced by non-binary employees.

## Introduction

Psychological violence and sexual harassment are critical challenges that human resource management (HRM) professionals must address to create safe and inclusive workplaces. This paper examines these issues from the perspective of non-binary employees, a group that is often overlooked in traditional HRM literature. Non-binary individuals are those whose gender identity does not fit within the binary categories of women and men. Despite the growing recognition of the importance of gender diversity in the workplace, there remains a scarcity of empirical research on the experiences of non-binary individuals, particularly from an HRM perspective (Lukkien et al., 2025; Ozturk et al., 2024). While some qualitative studies have begun to shed light on these experiences, quantitative research remains limited (for an exception see Davidson, 2016), often constrained by small sample sizes and methodological challenges (Beauregard et al., 2018; Humbert & Guenther, 2021; Ozturk & Tatli, 2016). Yet, HRM professionals need robust data to inform interventions that can effectively address the unique challenges faced by non-binary employees, particularly concerning psychological violence and sexual harassment, which are pressing issues in workplace management that are often left out of inclusion work.

This paper responds to this need for empirical evidence. It draws on a large-scale survey conducted in 15 countries across Europe with nearly 18,000 staff at universities and other research organisations, including 173 who identify as non-binary. By focusing on the experiences of non-binary individuals, the paper uses primary survey data to shed light on how HRM practitioners can better understand and mitigate the risks that this group faces in gendered organisational environments. A foundational body of work, originating with Acker’s (1990, 1992) work on the ‘gendered organisation’, has explored how organisations perpetuate gendered inequalities. While this literature has provided valuable insights, it often does so through a binary lens. Acker (2006, p. 146) herself often describes the gendered organisation in binary terms by pointing to socially structuring differences, for example explaining that ‘gendered’ means that ‘advantage and disadvantage, exploitation and control, action and emotion, meaning and identity, are patterned through and in terms of a distinction between male and female, masculine and feminine [sic]’. This binary framework overlooks the experiences of individuals who do not fit neatly into these categories, particularly non-binary people, thereby leaving a significant gap in both academic understanding and practical HRM strategies.

As a theoretical context and backdrop to the analysis, we therefore draw on the concept of the gendered organisation (Acker, 1990, 1992) to explain how workplaces structured around binary norms inherently fail to include non-binary individuals but also perpetuate systemic biases to those who do not conform to the gender binary. Organisational policies, practices, and cultures are often designed to reinforce traditional gender roles, marginalising those who do not conform to these norms (Ozturk et al., 2024). Further, cisnormative and binarist gender regimes are pervasive in HR practices, in turn restricting both acceptance of and support for non-binary employees in the workplace (Hennekam & Köllen, 2023). We argue that the negative experiences of non-binary individuals in gendered organisations are compounded by stigmatised identities, which lead to discrimination or social rejection in the workplace. Within gendered organisations, non-binary individuals are stigmatised due to their non-conformance to the binary gender norms. This stigma not only reinforces their marginalised status but also makes them more susceptible to negative workplace experiences such as sexual harassment and psychological violence. The stigma attached to their gender identity thus exacerbates the systemic disadvantages imposed by the binary gendered nature of organisations.

For non-binary individuals, the gendered organisation and associated stigma create a highly stressful environment. Individuals belonging to marginalised groups already experience unique, chronic stressors related to their minority status according to minority stress theory as originally developed by Meyer (2003). The frequent experiences of harassment and violence are significant stressors that add to the daily challenges of navigating a workplace that does not recognise or support their identity (Fletcher & Everly, 2021). This cumulative stress contributes to negative outcomes such as feeling unsafe, unwell, and socially excluded at work. We are therefore interested in the negative outcomes which are generated as a result, focusing our analysis on the extent to which different gender identity groups feel socially excluded, unsafe or unwell. We then examine how this relates to experiences of psychological violence and sexual harassment at work. In doing so, this paper contributes to a deeper understanding of how binary gendered organisational structures impact non-binary employees. We offer evidence-based recommendations to support HRM professionals in mitigating risks and promoting the well-being of all employees by addressing the problem of psychological violence and sexual harassment at work and regarding them as key EDI issues.

## Literature review and theoretical background

### Definitions and conflations

Inequalities at work is a topic that has generated much research, though some minoritised groups have been less studied than others (Fletcher & Swierczynski, 2023; Lukkien et al., 2025). While there is a large body of work looking at gendered experiences of work among women, the (gendered) experiences of LGBT (lesbian, gay, bisexual, transgender) employees have not been considered to the same extent (McFadden, 2015; Ragins, 2004). However, amidst the emerging body of work on the experiences of LGBT people at work lies a fundamental conceptual issue. The umbrella term ‘LGBT’ itself conflates experiences based on different inequality grounds: sexual orientation, trans status and gender identity (Fiani & Han, 2019). ‘Sexual orientation’ refers to an individual’s emotional, romantic, or sexual attraction to others (Fletcher & Marvell, 2023), which may include heterosexual, homosexual, bisexual, asexual, pansexual and other orientations. ‘Trans status’ indicates whether an individual identifies as transgender, meaning their gender identity differs from the sex assigned at birth. ‘Gender identity’ is an individual’s personal sense of being a woman, a man, both, neither, or anywhere along the gender spectrum (Fletcher & Swierczynski, 2023; Matsuno & Budge, 2017), which may not correspond with the sex assigned at birth. The distinction matters since these grounds are not necessarily aligned (Factor & Rothblum, 2008; Ozturk & Tatli, 2016) and the distinction is needed for ontological depth (Walby, 2007; Walby et al., 2012), ensuring that it is not assumed that different groups will necessarily face the same structural inequalities and discriminatory outcomes (Verloo, 2006). For example, while policies and practices exist within higher education institutions, they tend to treat all staff equally, without necessarily assessing the intersectional impacts of such policies and practices (Blell et al., 2023). Indeed, as Sawyer et al. (2013, p. 83) argue, it is ‘vital that we expand our focus to consider various identities at work in organizations and to remember that our end goal should always be to understand how these identities intersect with one another in meaningful ways’.

Trans people ‘disrupt and cross gendered norms and boundaries’ (Hennekam & Ladge, 2023, p. 7), but ‘crossing’ and ‘disrupting’ are not the same processes. Non-binary people disrupt the binary, but do not necessarily cross it. Matsuno and Budge (2017) emphasise that non-binary identities are diverse, with some individuals experiencing their gender as fluid, while others may see themselves as entirely outside the traditional binary categories. Additionally, non-binary individuals may experience a blend of masculine and feminine traits, or reject these labels entirely, highlighting the complexity and variability inherent in non-binary experiences. Being non-binary thus concerns one’s gender identity and expression, and though it may be related to sexual orientation and/or being trans, these are separate concepts (Fiani & Han, 2019). While some non-binary individuals identify as trans, there is not a direct alignment in many cases. Fletcher and Swierczynski (2023) for example state that between a third and a half of non-binary people also identify as trans. Conversely, Matsuno and Budge (2017) found that about a third of trans people identify as non-binary. Because of the distinctions between concepts, in this article, we focus specifically on gender identity, and the experience of non-binary people at work.

### LGBT experiences in the workplace

Bearing these conflations in terminology in mind, not all identities have been studied equally. There are, comparatively, more studies on LGB people while studies on or including trans people remain rare (McFadden, 2015; Webster et al., 2018), and for non-binary people even more so (Hennekam & Ladge, 2023) particularly in relation to workplace experiences (see Fletcher & Everly, 2021 for an exception). Indeed, a recent systematic review of intersectionality in EDI practices in the workplace does not include gender identity (Lukkien et al., 2025). These omissions are explained by challenges related to achieving sufficient sample sizes, connected with those who ‘pass’ and thus remain concealed, wishes to remain silent and below the radar (Beauregard et al., 2018; McFadden, 2015). LGB voices are thus more likely to prevail, compared to transgender and non-binary individuals who are often considered by cisgender people as ‘an obscure and misunderstood subgroup of the gay community’ (Curry, 2014, cited in Beauregard et al., 2018).

Overall, and despite evidence of resilience and coping strategies (Meyer, 2015), LGBT people have more negative experiences in the workplace, manifested in terms of lower job satisfaction, hindered career progression and development, unease at not being able to be their true authentic selves at work and concerns about discrimination and bias towards them (McFadden, 2015; Webster et al., 2018). Studies specifically on LGB people have shown that they were more likely to experience discrimination within and beyond the workplace because their sexual orientation represents an invisible stigma—similar to religion, class and hidden disabilities (Ragins, 2004). Stigmatised people evolve in a hostile and stressful social environment created by discrimination and prejudice. This additional stress, sometimes called ‘minority stress’, is a direct result of that stigmatised position (Meyer, 2003). Meyer identifies particular conditions that contribute to minority stress which include hiding and concealing one’s identity, expectations of rejection, the potential for violence and discrimination, and internalised homophobia. We find parallels with the challenges that non-binary people potentially come across, such as complexity of disclosure, identity incongruence, expectations of rejection and social exclusion, and the potential for violence and discrimination, all of which are likely to yield adverse outcomes.

Trans and non-binary people also face additional challenges and stigma stemming from how organisations are structured around binary categorisations of individuals’ gender and sexual orientation (Beauregard et al., 2018; Pringle, 2008) which permeate hierarchies, roles, processes, behaviours and scripts around gender and gender identity (Chapman & Gedro, 2009). In the space of a generation, many countries have decriminalised homosexuality, adopted same-sex marriage legislation, and provided adoption rights for same sex parents. However, progress is slow and uneven, and legal or organisational protection for trans people particularly patchy (Webster et al., 2018). Furthermore, legal protections for non-binary individuals remain inconsistent and are often explicitly excluded from existing equalities and anti-discrimination legislation, as non-binary identities remain often unrecognised (Matsuno & Budge, 2017). In many countries, it is still challenging or even impossible to have a legal non-binary gender marker on identification documents. This lack of recognition has significant implications for organisational policies and practices concerning gender, sex, and LGBT+ inclusion. Many organisations may focus primarily on legal compliance, which often does not encompass non-binary identities, considering them too difficult or legally ambiguous to address comprehensively. While some employers may adopt a general approach of inclusivity, this can miss critical nuances and specific needs of non-binary people.

Being non-binary continues to be a stigmatised identity, because it falls outside of the binary classification established under gender as a salient axis through which organisations and society are organised, and which creates an imperative to classify individuals into the binary categories of women and men (Acker, 2006). This is an automatic and largely unconscious process of ‘reading’ someone’s gender called ‘cognitive recognition’ (Goffman, 1963). According to Moulin de Souza and Parker (2022, p. 68) ‘binary thinking is an ontoepistemological strategy that shapes our representations, subjectivities and practices within organizations and beyond them’. Organisations are shaped by the hegemonic standard of men, white, heterosexual employees (Parker, 2002), who are positioned as normal or natural. Employees that do not conform or do not identify with this standard are positioned as ‘abnormal’ or ‘Other’ (Ahmed, 2006; Butler, 1990; Rumens, 2016). Workers need to align themselves with a cisnormative organisational space, where these spaces are invariably ‘straight’ and render these workers as ‘strangers’ or ‘bodies out of place’ (Ahmed, 2000, 2006). As such, ‘bodies which can’t or won’t align with these spaces will always be transgressive’ (Vitry, 2021, p. 936).

These ‘other’ bodies relate not only to that space, but also to the other bodies that occupy it. Inequalities in relation to gender identity thus need to be understood in terms of how organisations are structured to maintain the cisnormative binary order that shapes these bodies and spaces. Being out of place is thus likely to create unease, though it is fundamental to understand this as relational, and shaped by the reactions of others. Because the gender of a non-binary person may seem ambiguous, or fall outside these binary categories, a feeling of unease, discomfort or even anxiety is often generated (Beauregard et al., 2018; Matsuno & Budge, 2017; Ozturk & Tatli, 2016). At its worst, non-binary people can ‘incite confusion or even disdain’ (McFadden, 2015, p. 148), resulting in adverse experiences at work, including social exclusion, feeling unsafe or feeling unwell, which all represent inter-related stressors. Studies have showed that non-binary people can face difficulties at work, such as feeling compelled to change jobs or personal ones, such as impacts on mental health and wellbeing (Bockting et al., 2013; Budge et al., 2013; Budge et al., 2010; Clements-Nolle et al., 2006; Miller & Grollman, 2015; Whittle et al., 2007). This can nurture a climate and organisational environment where visibly non-binary people are most exposed, and may thus feel socially excluded, unsafe or unwell. This leads us to our first hypothesis:

H1: Non-binary people are more likely to feel socially excluded, unwell and unsafe at work.

Minority stress can be mitigated by ‘social safety’, which Diamond and Alley (2022, p. 2) define as the ‘basic human concern and connection [which] allow us to move through our social worlds without fear, because they remind us that we belong to an interconnected and protective social fabric’. Not only is this social fabric not available to non-binary people, due to their stigmatised identity, but it may be actively destroyed by manifestations of violence. After all, the opposite of safety is violence itself. Thus, if non-binary people cannot easily be typed within the binary gender order, not only does this create unease, but it can also result in even more problematic responses that amount to bullying and (sexual) harassment (Ozturk et al., 2024), just as those experienced by trans people (Rundall & Vecchietti, 2010; Whittle et al., 2008). Stigma therefore does not only put those that do not conform to the gender binary at risk of unfair treatment or discrimination, but also puts them at risk of violence (Webster et al., 2018).

A number of studies have reported a high proportion of LGBT people having experienced violence, and particularly sexual harassment within the workplace (Fletcher & Marvell, 2023; Grant et al., 2011; Hill & Silva, 2005; Konik & Cortina, 2008; Webster et al., 2018). Transgender people have been reported as the most highly targeted group experiencing physical and psychological violence (Beauregard et al., 2021; Grant et al., 2011; Rundall & Vecchietti, 2010; Witten, 2007). Sexual harassment can also be used as a strategy to police gender in the organisation, and to punish people that are seen to defy accepted gender norms and roles and assumed non-heterosexuality (Konik & Cortina, 2008; Rabelo & Cortina, 2014). Perversely, stigma against LGBT individuals is often used as a justification for psychological and sexual harassment (Brassel et al., 2019). Focusing on sexual harassment and psychological violence is essential due to their widespread prevalence and adverse consequences on employees (Humbert & Strid, 2024). Addressing these challenges is not only a legal and ethical obligation for organisations but also crucial for fostering a safe, inclusive, and supportive workplace culture. Accordingly, we hypothesise the following:

H2: Non-binary people are more likely to experience psychological violence and sexual harassment at work.

H3: Experiences of psychological violence and sexual harassment will mediate the relationship between being non-binary and feeling socially excluded, unsafe and unwell at work.

**Figure 1.**
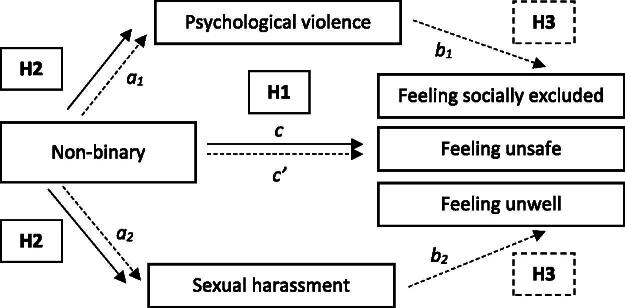
Hypotheses.

Our conceptual model is summarised in Figure 1. This paper contributes empirically and conceptually to diversity and human resource management research, arguing that it has overlooked the specific experiences of non-binary individuals despite scholarly interest and literature in sexual orientation and gender identity research (Beauregard et al., 2018; Bell et al., 2011; Ozturk & Tatli, 2016; Ragins et al., 2007). The broad research questions examined are: What potential adverse outcomes do non-binary people face in relation to feeling excluded, feeling unwell, feeling unsafe? Are these adverse outcomes related to experiences of gender-based violence, notably psychological violence and sexual harassment, at work?

## Methodology

### Study context

This study was conducted within institutions in the higher education sector, which present a unique occupational context for understanding issues related to gender identity, sexual orientation, and workplace inequalities. Since 2022, it has become mandatory for higher education institutions to have a Gender Equality Plan in place to remain eligible for EU research funding (European Commission, 2021). However, even though gender-based violence is one of the recommended areas, few institutions focus on this in policy or practice (European Commission, 2024). Moreover, while there is an increasing focus on inclusive gender equality plans, they focus on EDI characteristics in conjunction with gender such as sexual orientation and gender identity but they do not approach intersectional issues comprehensively (Mueller & Humbert, 2025). Even where policies and measures are in place, their effectiveness is frequently questioned due to inconsistent implementation, insufficient enforcement, and cultural barriers within institutions that may undermine these initiatives (Huck et al., 2022). Additionally, tensions exist regarding the extent to which these measures address the needs of non-binary individuals, who are often overlooked or explicitly excluded from institutional frameworks. While this study is situated within the higher education sector, its findings have relevance for organisations more widely. Many workplaces face similar challenges in bridging the gap between formal diversity policies and their practical implementation (Colgan et al., 2007; Webster et al., 2018), particularly concerning the inclusion and protection of LGBTQ+ employees, where the presence of supportive colleagues and supervisors plays a crucial role in fostering a genuinely inclusive environment (Fletcher & Swierczynski, 2023; Schönauer et al., 2025). The insights gained from this context highlight broader organisational dynamics, such as the impact of systemic biases, the importance of inclusive practices, the prevalence of gender-based violence and the need to address minority stress across different sectors.

### Description of survey

This article draws on the online UniSAFE Survey: Gender-based violence and institutional responses (Lipinsky et al., 2022), carried out in 15 European countries in the first half of 2022, in 46 European universities and other research organisations. Ethics approval for the survey was granted by GESIS and the Swedish Ethical Review Authority. The survey is a quasi-census of all staff and students at these institutions and collected *n* = 42,186 responses (Lipinsky et al., 2022; Schredl et al., 2023). Because of the organisational lens applied in this article, the analysis is limited to staff, representing *n* = 17,993 respondents. Although the proportion of people that identify as non-binary or with another gender identity than the binary categories of women and men is small—a reason often cited for excluding minoritised gender identity groups from research studies (Humbert & Guenther, 2021)—the relatively large number of respondents provides a sufficient cell count for analysis. In total, 17,951 staff provided information on their gender identity: *n* = 173 identified as ‘non-binary’, *n* = 11,624 as ‘women’, *n* = 6024 as ‘men’, and *n* = 130 with a gender identity ‘not listed’ (this is a heterogeneous group and is included for transparency rather than as a focal point of the analysis).

The questionnaire was developed through a rigorous, multi-step process to ensure its robustness and relevance. Initially, a scoping review was conducted to identify existing measures and frameworks (Schredl et al., forthcoming). This scoping review provided a basis for drafting the initial version of the questionnaire. To further enhance the quality and validity of the questionnaire, the initial draft underwent thorough expert review, focusing specifically on content validity. Experts evaluated the questionnaire’s items, assessing their relevance and alignment with the underlying constructs. Following this, the questionnaire was pretested to evaluate its clarity, relevance, and comprehensiveness in capturing the targeted issues. Feedback from this pretesting phase informed refinements to improve readability, appropriateness, and ease of understanding across diverse participant demographics (Lipinsky et al., 2021). Given the multinational scope of the project, it was essential to ensure linguistic and cultural inclusivity and accordingly the questionnaire was translated into 14 languages, with each translation reviewed by 2–3 experts to verify accuracy and cultural relevance (Schredl et al., 2023).

Weights were calculated using data from the participating organisations on their sex composition, proportion of academic and non-academic staff and proportion working in a STEMM field (Schredl et al., 2023). Weights were not computed for institutions with fewer than 100 responses (*n* = 5), or which did not provide administrative data (*n* = 1). Weights are available for 98.6% of observations and the remaining are assigned a weight of 1. After weighting, the sample consisted of 0.96% of people identifying as non-binary, 48.44% as women, 49.87% as men and 0.73% with another gender identity not listed in the survey.

### Measures

Characteristics related to demographic and functional diversity are considered in the analysis. Demographic diversity includes variables such as ethnicity or age, while functional diversity examines organisational roles, such as occupation or contractual arrangements (see Table 1 for the variables and categories included). For completeness, we present results for people that identify with another gender identity, though because of the difficulty in unpacking the category, we do not perform a detailed analysis.

**Table 1.** Descriptive statistics – categorical variables.

		Total	A gender identity not listed	Men	Non-binary	Women	*p*
		%	%	%	%	%	
*n* = 17,812	A gender identity not listed	0.66					
	Men	49.94					
	Non-binary	0.93					
	Women	48.47					
*n* = 17,638	Cis	99.40	85.85	99.72	51.32	99.82	<.01***
	Trans	0.60	14.15	0.28	48.68	0.18	
*n* = 16,996	Asexual	0.90	:	0.71	4.97(u)	0.97	<.01***
	Bisexual	4.19	14.04	3.25	16.15	4.84	
	Heterosexual	89.73	39.98	89.46	25.05	91.72	
	Homosexual	3.64	:	5.80	13.72	1.25	
	Queer	0.89	15.45	0.36	29.17	0.78	
	A sexual orientation not listed	0.65	25.54	0.42	10.95	0.44	
*n* = 17,441	Disability or chronic illness	11.28	29.10	10.13	26.07	11.99	<.01***
	No disability nor chronic illness	88.72	70.90	89.87	73.93	88.01	
*n* = 17,587	Minority ethnic background	4.66	19.76	5.00	16.80	3.92	<.01***
	Non-minority ethnic background	95.34	80.24	95.00	83.20	96.08	
*n* = 17,812	Academic staff	56.47	70.48	63.16	61.08	49.30	<.01***
	Non-academic staff	43.53	29.52	36.84	38.92	50.70	
*n* = 17,812	Fixed-term contract	28.21	32.07	27.15	49.49	28.84	.03**
	Permanent contract	71.79	67.93	72.85	50.51	71.16	

Weighted percentages, *n* represents the unweighted number of observations. *p* value based on χ^2^ test. Cell counts of 5 or below are not reported, and marked with ‘:’. Cell counts above 5 but below 10 are unreliable due to small cell count, and marked with ‘(u)’. ** *p* < .05; *** *p* < .01.

A range of potential adverse consequences were used as dependent variables, capturing the extent to which respondents felt socially excluded, unsafe or unwell. Feeling socially excluded was measured through four items (*α* = 0.8139), feeling unsafe through 16 items (*α* = 0.6483), and feeling unwell through seven items (*α* = 0.8913), detailed in Tables 2–4. All items were presented in random order. The responses to items in the first two instances were dichotomous, coded as 0 for ‘no’, and 1 for ‘yes’. For the third, the response consisted of four categories (Never; Once; 2–5 times; 6 times or more), and was recoded into a dichotomous variable, with 0 for ‘none or up to 5 times’ and 1 for ‘6 times or more’ so as to capture the most severe manifestations of feeling unwell.

**Table 2.** Descriptive statistics – numerical variables.

			Mean	SD	*p*
*n* = 17,474	Age in years	Total	44.88	11.11	<.01***
*n* = 106		A gender identity not listed	44.47	10.98	
*n* = 5894		Men	45.24	11.34	
*n* = 164		Non-binary	39.78	11.22	
*n* = 11,310		Women	44.61	10.83	
*n* = 17,812	Time in organisation in years	Total	12.95	10.55	<.01***
*n* = 116		A gender identity not listed	11.83	9.22	
*n* = 5987		Men	13.50	10.82	
*n* = 166		Non-binary	9.75	10.09	
*n* = 11,543		Women	12.47	10.26	

Weighted summary statistics, *n* represents the unweighted number of observations. *p* value based on ANOVA. ** *p* < .05; *** *p* < .01.

**Table 3.** Feeling socially excluded by gender identity.

Since you started at your institution, have you ever been in a situation where someone…	Total	Gender identity not listed	Men	Non-binary	Women		
	%	%	%	%	%	*n*	*p*
…put you down or was condescending to you in some way?	39.89	60.04	31.32	50.61	48.20	15,053	<.01***
…paid little attention to a statement you made or showed little interest in your opinion?	47.92	62.69	41.76	69.05	53.63	15,063	<.01***
… ignored or excluded you from the group or team?	27.31	43.06	21.17	45.50	33.07	15,059	<.01***
… ignored you or failed to speak to you?	40.31	53.14	33.67	55.84	46.60	15,093	<.01***
One or more types of social exclusion	60.89	74.76	53.73	75.18	67.70	15,327	<.01***

Weighted percentages, *n* represents the unweighted number of observations. *p*value based on χ^2^ test. *** *p* < .01.

**Table 4.** Feeling unsafe by gender identity.

Since you started at your institution, have you felt unsafe in any of the following spaces?	Total	A gender identity not listed	Men	Non-binary	Women		
	%	%	%	%	%	*N*	*p*
Break room, canteen or cafeteria	2.83	7.20(u)	2.11	6.03(u)	3.43	14,631	<.01***
Classroom, lecture theatre, seminar or meeting room	5.49	10.95(u)	4.42	11.21	6.38	14,631	<.01***
Library	0.52	:	0.36	:	0.59	14,631	.01***
In the lab or a staff office	9.39	9.57(u)	7.17	19.95	11.41	14,631	<.01***
While out conducting fieldwork	1.53	:	1.23	:	1.81	14,631	.08
Residential accommodation	0.25	:	0.20	:	0.30	14,631	.48
Toilets	1.16	:	0.52	7.11	1.67	14,631	<.01***
Lift, stairs or corridor	3.81	:	1.70	4.33(u)	5.90	14,631	<.01***
Multi-storey car park	2.48	:	1.03	:	3.92	14,631	<.01***
Outdoor spaces in the institution’s premises	3.50	:	1.73	4.15(u)	5.26	14,631	<.01***
At a conference	2.21	8.30(u)	1.30	11.91	2.87	14,631	<.01***
In connection with a study or work-related activity in the evening	3.04	:	1.67	6.32(u)	4.31	14,631	<.01***
In connection with an activity not related to study or work in the evening but connected to your institution	1.73	:	1.10	:	2.34	14,631	<.01***
Online, e.g. threats *via* social media, email, messages, or virtual learning platforms	3.36	:	2.79	10.46	3.75	14,631	.01***
A gym or sports facility that is part of your institution	0.23	:	0.11(u)	:	0.34	14,631	.04**
At another place or in a situation other than those listed above	3.41	:	2.57	4.42(u)	4.22	14,631	.01***
One or more types of unsafe places	24.29	28.61	16.73	43.87	31.42	14,631	.01***

Weighted percentages, *n* represents the unweighted number of observations. *p* value based on χ^2^ test. Cell counts of 5 or below are not reported, and marked with ‘:’. Cell counts above 5 but below 10 are unreliable due to small cell count and marked with ‘(u)’. ** *p* < .05; *** *p* < .01.

Psychological violence and sexual harassment were measured using seven and six, respectively, randomised items (*α* = 0.8147 and *α* = 0.7967) and have been validated in Humbert et al. (2022). The list of items is provided in Tables 5 and 6. Because of the potentially triggering nature of the questions, respondents could opt to check a box to allow them to skip these questions and continue the survey. Responses were dichotomous, coded as 0 for ‘no’ and 1 for ‘yes’. The overall prevalence of psychological violence and sexual harassment, respectively, was calculated as having responded ‘yes’ to at least one incident, divided by the total number of respondents who did not skip the question.

**Table 5.** Feeling unwell by gender identity.

These questions are about how you have been in the past three months. How often have you… (6 times or more)	Total	A gender identity not listed	Men	Non-binary	Women		
	%	%	%	%	%	*N*	*p*
… had stomach ache, headache or tension in various muscles?	24.51	34.00	17.76	37.61	31.01	15,180	<.01***
… been physically exhausted?	29.18	42.69	23.24	41.30	34.83	15,185	<.01***
… slept badly or restlessly or found it hard to go to sleep?	34.60	40.60	29.92	40.66	39.17	15,195	<.01***
… been emotionally exhausted or felt worn out?	29.51	52.97	24.05	41.63	34.55	15,172	<.01***
… been irritable or tense?	24.33	39.47	19.77	36.41	28.56	15,156	<.01***
… had problems to concentrate?	27.16	35.13	23.69	37.57	30.40	15,140	<.01***
… felt sad or guilty?	18.58	39.06	15.02	27.90	21.80	15,065	<.01***
One or more symptoms of physical or mental health	54.55	66.13	48.34	69.77	60.43	15,314	<.01***

Weighted percentages, *n* represents the unweighted number of observations. *p* value based on χ^2^ test. *** *p* < .01.

**Table 6.** Experiences of psychological violence and sexual harassment by gender identity.

Since you started at your institution, has someone ever done any of the following to you?	Total	A gender identity not listed	Men	Non-binary	Women		
	%	%	%	%	%	*N*	*p*
Psychological violence							
Directed abusive comments towards you (e.g. demeaning, humiliating, offensive or ridiculing comments)	29.77	39.71	24.73	40.22	34.56	17,411	<.01***
Made threatening comments towards you	15.41	25.42	14.77	20.98	15.84	17,411	<.01***
Gave you hostile looks, stares, or sneers	30.77	34.79	27.10	42.46	34.21	17,411	<.01***
Interrupted you, spoke over you or addressed you in disrespectful terms in front of others	46.49	54.86	39.87	61.75	52.80	17,411	<.01***
Unfairly rated you lower than you deserve in an evaluation or assessment	24.60	32.70	20.39	32.05	28.64	17,411	<.01***
Ignored you or did not speak to you	45.04	53.27	39.42	58.75	50.37	17,411	<.01***
Subjected you to an outburst of anger	38.24	44.45	33.06	40.66	43.37	17,411	<.01***
One or more incidents of psychological violence	68.37	69.48	62.66	80.94	73.90	17,411	<.01***
Sexual harassment							
Asked intrusive questions about your private life	14.28	28.42	9.65	24.93	18.60	16,076	<.01***
Stared or leered inappropriately at you	12.25	10.27	3.83	18.37	20.64	16,076	<.01***
Made sexually suggestive comments or jokes	23.74	28.04	16.52	33.82	30.80	16,076	<.01***
Made intrusive comments about your physical appearance	14.48	21.55	7.43	29.68	21.23	16,076	<.01***
Made inappropriate invitations to go out on dates	5.35	10.48	2.22	5.75	8.45	16,076	<.01***
Touched, hugged or kissed you in an unwelcome manner	7.05	11.97	2.97	10.74	11.04	16,076	<.01***
One or more incidents of sexual harassment	33.59	38.95	23.49	47.91	43.44	16,076	<.01***

Weighted percentages, *n* represents the unweighted number of observations. *p* value based on χ^2^ test. *** *p* < .01.

### Analytical approach

Structural inequalities are shaped by gender, but also other identities that together create multiple and intersectional forms of discrimination and disadvantage. Intersectional inequalities can be understood as shaped by different axes of power among various sets of social relations (Walby et al., 2012), and taking an intersectional approach means looking at different positionalities. However, reconciling the tenets of intersectionality theory with the needs of a quantitative approach is complex. Taking a categorical approach risks over-stabilising groups and thereby essentialise and reify differences and social relations (Hancock, 2007; McCall, 2005; Walby et al., 2012). Temporarily stabilising categories is necessary for empirical analysis, and in this article, we regard intersectional categories as ‘heuristic devices’, that is as tools that allow for an analysis of structural inequalities at work (Cho et al., 2013).

In this paper, structural inequalities are therefore an integral component of the analytical framework. This approach reflects the argument that quantitative analyses of social inequalities must systematically account for structural and intersectional dimensions, much like economic models control for structural factors such as GDP per capita. By treating intersectional categories as heuristic tools, this study demonstrates how structural inequalities can be analysed quantitatively in a way that respects the complexity of intersectional relations and ensures that structural inequalities are at the centre of the analysis, emphasising their role in shaping differential experiences and outcomes for non-binary people at work.

We draw on scholarship that transposes intersectionality into quantitative analysis using multi-level modelling (Bauer et al., 2021; Evans et al., 2018; Humbert, 2024; Merlo, 2018). Multilevel modelling provides a robust framework for analysing data with a hierarchical or nested structure, which is particularly suited for addressing intersectional inequalities. This approach allows us to account for both individual-level characteristics and the broader social contexts or groups to which individuals belong. For instance, individuals may be nested within groups defined by intersectional categories such as gender identity, ethnicity, and workplace. By incorporating random effects, multilevel modelling captures the variability between these groups, while fixed effects identify consistent patterns across the dataset. This ensures that the heterogeneity within and between groups is explicitly modelled, offering a more nuanced understanding of how structural inequalities operate. In the context of intersectionality, multilevel modelling avoids the pitfalls of traditional methods by recognising the complexity of overlapping social identities and their impact on experiences of inequalities.

Intersectional inequalities are modelled by including all possible intersectional strata to capture how individuals may share similar experiences with their intersectional membership group (Evans et al., 2018). Intersectional strata are generated according to seven variables: gender identity (4 possible responses); gender currently the same as sex assigned at birth (2); age groups in 5-year bands (10); staff type (2); disability or chronic illness (2); minority ethnic group (2); sexual orientation (6). This creates a total of 586 non-empty strata. The multi-level models are also able to account for how experiences might relate to the different organisations and countries in which people work and live. We use a cross-classified multi-level model (Leckie, 2013) consisting of three levels: countries (level 4); organisations (level 3); and intersectional strata (level 2). As our dependent variables are dichotomous, we use a logit link function.

The use of multilevel modelling is also particularly suited for datasets with unbalanced group sizes, as the estimates for each group are ‘shrunk’ toward the overall mean, allowing us to mitigate the risk of overestimating effects in smaller groups like those for non-binary or other gender identities. This therefore allows for the inclusion of small subgroups (such as non-binary and other gender identities) without compromising the robustness of the overall model. We present both a reduced and a full model to comprehensively understand the dynamics of gender identity and its entanglement with other social identities. The reduced model focuses on the main effect of being non-binary alone. The full model, in contrast, goes beyond the individual effect of being non-binary by examining how gender identity is entangled with other axes of inequalities, such as sexual orientation. The models are fitted through the external software package ‘runmlwin’ (Charlton et al., 2024; Leckie & Charlton, 2012) within Stata v18, using the IGLS (Iterative Generalised Least Squares) algorithm and PQL2 method.

The mediation analysis follows Preacher and Hayes (2004, 2008) recommendations to generate bootstrap estimates of path coefficients. The analysis relies on 5,000 iterations. In all models, the number of years spent working at the institution is used as a control variable. Since individuals with longer tenures have greater potential to experience violence, tenure represents an important contextual factor that could confound the relationship between gender identity and experiences of violence. This aligns with the purpose of accounting for other meaningful variables, as described by Carlson and Wu (2012), wherein control variables are used to isolate the focal relationship by addressing confounding influences. Following their recommendation to align control variable use with a clearly stated intent, tenure is included to ensure that the results reflect differences attributable to gender identity rather than disparities in exposure time. This approach ensures greater interpretability and validity of the findings.

## Results

### Descriptive analysis

The sample characteristics are provided in Tables 1 and 2. Gender identity is strongly associated with being cis or trans (*p* < .01). Nearly all who identify as women and men are cis. However, this only applies to about half of those that identify as non-binary, and 86% of those who identify with another gender identity. The sexual orientation of people who identify within or beyond the binary differs markedly (*p* < .01). Most people who identify as women and men—about nine in ten—say they are heterosexual, compared to one in four non-binary people and two in five people who identify with another gender identity.

Non-binary people and people identifying with another gender identity are much more likely to report having a disability or chronic illness (*p* < .01). This concerns over one in four of those identifying as non-binary or with another gender identity, compared to just over one in ten of those identifying as women or men. Non-binary people and people identifying with another gender identity are also much more likely to belong to a minority ethnic group (*p* < .01). Nearly one in five of those that identify as non-binary or with another gender identity say they belong to a minority ethnic group, in contrast to less than one in twenty people identifying as women or men. Non-binary staff are younger on average (*p* < .01), with a mean age just below 40 years, compared with about 45 years for the other gender identity groups.

Non-binary staff have also spent less time on average working in their organisation (*p* < .01), with fewer than 10 years on average, compared with 12 years or more for the other gender identity groups. Women are over-represented in non-academic staff (*p* < .01). Women represent only about half of academic staff, compared to about two in three (or more) for other gender identity groups. This corresponds to a well-known pattern of occupational segregation, where women are over-represented in less prestigious occupations (Acker, 1990), though interestingly it does not seem to affect people who identify with a gender outside the binary. Non-binary people are less likely (*p* < .05) than other gender identity groups to hold a permanent contract (50% compared to approximately 70% for all other groups).

All forms of social exclusion differ by gender identity (Table 3). Men are systematically less affected, and the proportion of women is higher in comparison (*p* < .01). Yet, for non-binary people and those with another gender identity, social exclusion is even higher. About 75% of those who identify as non-binary or with another gender identity report having experienced at least one form of social exclusion. This compares to about two-thirds of women (68%) and over half of men (54%).

Being non-binary is associated with a much greater likelihood of feeling unsafe (*p* < .01) (Table 4). Over four in ten non-binary people (44%) felt unsafe in one or more places linked to their organisation. Approximately one in three people that identify with another gender identity (29%) and women (31%) feel unsafe in at least one location. Men are least likely to feel unsafe in any location (17%). The places were non-binary people felt the most unsafe, relative to other gender groups, were in the lab or staff office (20%), at conferences (12%) or in classrooms or other teaching spaces (11%). Feeling unsafe online was also considerably higher for non-binary people (10%) compared to women and men. Importantly, toilets were also perceived as unsafe by a much higher proportion of non-binary people (7%) compared with women and men.

Feeling unwell, as measured by experiences of psychosocial risks, shows that men are the least affected gender identity group (*p* < .01) (Table 5). Over half of respondents (55%) have experienced one or more symptoms of physical or mental health six times or more in the three months prior to the survey, however, this is highest among non-binary people (70%) and people identifying with another gender identity (66%). For example, non-binary people and people who identify with another gender identity are more likely than any of the other gender groups to experience emotional exhaustion or feeling worn out (42% and 53% respectively) six times or more, or to experience physical exhaustion (41% and 43% respectively) six times or more.

Being non-binary is associated with a systematic higher prevalence of psychological violence (*p* < .01), except for being subjected to an outburst of anger which also disproportionately affects women and people who identify with another gender identity, compared with men (Table 6). Over four in five (81%) non-binary people report having experienced one or more incidents of psychological violence since starting at their institution. The most prevalent forms of psychological violence for non-binary people include being interrupted, spoken over or addressed in disrespectful terms in front of others (62%), being ignored or not spoken to (59%), or receiving hostile looks, stares, or sneers (42%).

Non-binary people also report more experiences of sexual harassment (*p* < 0.01), with nearly half (48%) having experienced one or more incidents of sexual harassment since starting in their institution (Table 6). This includes, for example, receiving intrusive comments about their physical appearance (30%) or being asked intrusive questions about their private life (25%). Gender harassment and heterosexism (Konik & Cortina, 2008) is apparent in that fewer non-binary people received inappropriate invitations to go out on dates (6%) compared with women (8%) or people who identify with another gender identity (10%).

### Multivariate analysis

The reduced models (controlling for time at the organisation, and taking into account the non-independence of experiences across intersectional strata, countries and organisations), all suggest that non-binary people face more adverse consequences in the workplace (Table 7). The estimates for the total effect (path c) suggest that non-binary people are nearly 6 times (e^β^ = 5.763, *p* < .01) as likely as men (used as the reference category as the most ‘privileged’ group) to feel socially excluded, nearly 6 times as likely (e^β^ = 5.851, *p* < .01) as likely to feel unsafe, and twice as likely (e^β^ = 2.025, *p* < .01) to feel unwell. The full models (adding variables for socio-demographic and functional diversity) confirm these findings for feelings of social exclusion (non-binary people are more than four times as likely as men to feel socially excluded, e^β^ = 4.452, *p* < .01) and feeling unsafe (non-binary people are about three times as likely as men to feel unsafe, e^β^ = 3.671, *p* < .01). However, while the reduced model suggests non-binary people were more likely to feel unwell, compared to men, this effect disappears when further intersectional factors are included. This suggests that being non-binary intersects with other diversity grounds, such as for example having a disability: 23% of non-binary people report having a disability or chronic illness, compared with 12% of women and 10% of men. H1 is therefore supported for feeling socially excluded and feeling unsafe, but not for feeling unwell.

**Table 7.** Mediation analysis for non-binary respondents: exponentiated means (e^β^) and 95% confidence intervals (CI) of the bootstrap estimates.

		Reduced model				Full model			
		**Feeling socially excluded**							
		e^β^	95% CI			e^β^	95% CI		
H1	Path c	5.763	3.192	10.406	***	4.452	2.118	9.356	***
H2	Path a_1_	4.450	2.458	8.056	***	2.434	1.181	5.019	**
	Path a_2_	5.424	3.210	9.165	***	3.010	1.602	5.653	***
	Path b_1_	14.592	12.949	16.443	***	14.463	12.808	16.331	***
	Path b_2_	3.042	2.724	3.397	***	3.026	2.713	3.374	***
H3	Path c’	3.103	1.607	5.990	***	3.318	1.531	7.189	***
		**Feeling unsafe**							
		e^β^	95% CI			e^β^	95% CI		
H1	Path c	5.851	3.578	9.567	***	3.671	1.956	6.890	***
H2	Path a_1_	4.422	2.456	7.962	***	2.434	1.200	4.935	**
	Path a_2_	5.415	3.206	9.145	***	3.015	1.608	5.653	***
	Path b_1_	4.695	4.064	5.424	***	4.576	3.953	5.298	***
	Path b_2_	3.156	2.832	3.518	***	3.104	2.785	3.459	***
H3	Path c’	3.976	2.353	6.718	***	3.031	1.570	5.852	***
		**Feeling unwell**							
		e^β^	95% CI			e^β^	95% CI		
H1	Path c	2.025	1.219	3.362	**	0.834	0.449	1.548	
H2	Path a_1_	4.414	2.452	7.945	***	2.449	1.184	5.066	**
	Path a_2_	5.411	3.242	9.031	***	3.037	1.641	5.619	***
	Path b_1_	1.963	1.785	2.159	***	1.943	1.763	2.141	***
	Path b_2_	1.811	1.652	1.985	***	1.747	1.591	1.918	***
H3	Path c’	1.497	0.911	2.462		0.690	0.376	1.266	

** *p* < .05; *** *p* < .01. Paths c represent the total effects of being non-binary on the outcome variables. Paths a_1_ and a_2_ refer to the effects of being non-binary on experiences of psychological violence and sexual harassment, respectively. Paths b_1_ and b_2_ capture the effects of experiences of violence on the outcome variables. This step allows for the calculation of paths c’, which represents the direct effects of being non-binary on the outcomes, after accounting for the mediating effects of experiences of violence. The reference category for gender identity is men.

The reduced model suggests that non-binary people are more likely to experience psychological violence (path a_1_) and sexual harassment (path a_2_). Non-binary people are over four times as likely as men to experience psychological violence (e^β^ = 4.450, *p* < .01; e^β^ = 4.422, *p* < 0.01; e^β^ = 4.414, *p* < .01 across the three dependent variables) and over five times as likely as men to experience sexual harassment (e^β^ = 5.424, *p* < .01; e^β^ = 5.415, *p* < .01; e^β^ = 5.411, *p* < .01). As expected, these values are very similar as the bootstrap estimates relate to the same underlying model. The full model shows that being non-binary continues to be associated with a higher risk of experiencing psychological violence (e^β^ = 2.434, *p* < .05; e^β^ = 2.434, *p* < .05; e^β^ = 2.449, *p* < .05) and sexual harassment (e^β^ = 3.010, *p* < .01; e^β^ = 3.015, *p* < .01; e^β^ = 3.037, *p* < .01), though the estimates have decreased in size compared to the reduced model, suggesting that both experiences of psychological violence and sexual harassment are also related to other grounds of inequalities. This might be because being trans or from a minoritised sexual orientation is entangled with gender identity. Among non-binary people, 16% are bisexual and 14% are homosexual, compared with 4% for each respectively among all respondents (*p* < .01). Even more strikingly, 48% of non-binary people are trans, defined widely as their current gender not being the same as sex attributed at birth, compared to less than 1% overall (*p* < .01). Hypothesis 2 is therefore supported.

We now look at the extent to which adverse consequences at work are mediated by experiences of psychological violence and sexual harassment. The analysis above has shown that non-binary people are more likely to experience sexual harassment and psychological violence (paths a_1_ and a_2_). The results establish that experiences of psychological violence and sexual harassment are positively associated with feeling socially excluded, unsafe and unwell (paths b_1_ and b_2_). Having experienced psychological violence is associated with being 14 times as likely to feel social excluded as those who have not (e^β^ = 14.592, *p* < .01 for the reduced model; e^β^ = 14.463, *p* < .01 for the full model), being over four times as likely to feel unsafe (e^β^ = 4.695, *p* < .01 for the reduced model; e^β^ = 4.576, *p* < .01 for the full model), and being nearly twice as likely to feel unwell (e^β^ = 1.963, *p* < .01 for the reduced model; e^β^ = 1.943, *p* < .01 for the full model). Similarly, having experienced sexual harassment is associated with being over three times as likely to feel social excluded (e^β^ = 3.042, *p* < .01 for the reduced model; e^β^ = 3.026, *p* < .01 for the full model), three times as likely to feel unsafe (e^β^ = 3.156, *p* < .01 for the reduced model; e^β^ = 3.104, *p* < .01 for the full model), and nearly twice as likely to feel unwell (e^β^ = 1.811, *p* < .01 for the reduced model; e^β^ = 1.747, *p* < .01 for the full model).

The direct effects of being non-binary (path c’) show a positive relationship with feeling socially excluded and feeling unsafe in both the reduced and full models. Being non-binary, accounting for the mediating effects of experiences of psychological violence and sexual harassment, is associated with being over three times as likely to feel socially excluded (e^β^ = 3.103, *p* < .01 for the reduced model; e^β^ = 3.318, *p* < .01 for the full model) and feeling unsafe (e^β^ = 3.976, *p* < .01 for the reduced model; e^β^ = 3.031, *p* < .01 for the full model). However, for feeling unwell, this relationship is not statistically significant. These results provide support for Hypothesis 3. These mediations illustrate the important role of experiences of sexual harassment and psychological violence in explaining why non-binary people are more likely to feel socially excluded, unsafe or unwell in their organisations. A possible interpretation is that part of feeling socially excluded and unsafe can be attributed to the overt victimisation of non-binary people, and their experiences of gender-based violence at work.

To aid interpretation and allow for comparison across gender identities, Table 8 presents the coefficient estimates for women, using men as the reference category. These results complement the earlier models focused on non-binary respondents. Looking at paths c, it appears that women are also more likely to feel social excluded and unsafe than men, but to a lesser extent than non-binary respondents. In the reduced model, both women and non-binary individuals were significantly more likely than men to report feeling unwell, with the association being stronger for non-binary respondents. However, this pattern shifts in the full model, which includes additional socio-demographic and structural characteristics. Once these intersectional factors are accounted for, the association between non-binary identity and feeling unwell is no longer statistically significant, while the effect remains for women. This suggests that the elevated likelihood of poor health among non-binary respondents is largely attributable to intersecting inequalities rather than gender identity alone. By contrast, for women, the association persists even after adjusting for these factors, indicating that gendered patterns of health disadvantage are more robust in this group.

**Table 8.** Mediation analysis for women respondents: exponentiated means (e^β^) and 95% confidence intervals (CI) of the bootstrap estimates.

		Reduced model				Full model			
		**Feeling socially excluded**							
		e^β^	95% CI			e^β^	95% CI		
H1	Path c	2.235	2.003	2.495	***	2.235	1.999	2.499	***
H2	Path a_1_	2.148	1.934	2.385	***	2.156	1.935	2.402	***
	Path a_2_	3.613	3.191	4.091	***	3.498	3.108	3.937	***
	Path b_1_	14.592	12.949	16.443	***	14.463	12.808	16.331	***
	Path b_2_	3.042	2.724	3.397	***	3.026	2.713	3.374	***
H3	Path c’	1.500	1.334	1.685	***	1.515	1.349	1.702	***
		**Feeling unsafe**							
		e^β^	95% CI			e^β^	95% CI		
H1	Path c	3.085	2.718	3.502	***	3.150	2.766	3.586	***
H2	Path a_1_	2.147	1.929	2.389	***	2.160	1.936	2.410	***
	Path a_2_	3.609	3.197	4.074	***	3.496	3.107	3.934	***
	Path b_1_	4.695	4.064	5.424	***	4.576	3.953	5.298	***
	Path b_2_	3.156	2.832	3.518	***	3.104	2.785	3.459	***
H3	Path c’	2.231	1.962	2.536	***	2.320	2.028	2.655	***
		**Feeling unwell**							
		e^β^	95% CI			e^β^	95% CI		
H1	Path c	1.769	1.594	1.964	***	1.806	1.619	2.015	***
H2	Path a_1_	2.147	1.929	2.390	***	2.158	1.937	2.403	***
	Path a_2_	3.617	3.201	4.087	***	3.501	3.108	3.945	***
	Path b_1_	1.963	1.785	2.159	***	1.943	1.763	2.141	***
	Path b_2_	1.811	1.652	1.985	***	1.747	1.591	1.918	***
H3	Path c’	1.465	1.319	1.627	***	1.508	1.350	1.683	***

*** *p* < .01. Paths c represent the total effects of being a woman on the outcome variables. Paths a_1_ and a_2_ refer to the effects of being a woman on experiences of psychological violence and sexual harassment, respectively. Paths b_1_ and b_2_ capture the effects of experiences of violence on the outcome variables. This step allows for the calculation of paths c’, which represents the direct effects of being a woman on the outcomes, after accounting for the mediating effects of experiences of violence. The reference category for gender identity is men.

In the reduced models, both women and non-binary individuals are significantly more likely than men to report experiences of psychological violence and sexual harassment, with non-binary people facing the highest risks. However, when intersectional characteristics are included, the differences between the two groups narrow. In the full models, both women and non-binary individuals remain significantly more likely than men to experience gender-based violence, but the estimated effects are now of a similar magnitude. This suggests that the higher risk observed for non-binary individuals in the reduced models is partly explained by their greater exposure to other forms of inequalities, factors also associated with increased vulnerability to violence. Once these are accounted for, the gender-based differences become more aligned, indicating that it is the accumulation of intersecting disadvantages, rather than gender identity alone, that shapes exposure to violence in the workplace.

After accounting for the mediating effects of psychological violence and sexual harassment, the direct effects (paths c′) show that non-binary individuals remain the most affected group in relation to social exclusion across both the reduced and full models. For feeling unsafe, non-binary people also report the highest levels in the reduced model. However, when intersectional characteristics are included, the difference between non-binary individuals and women narrows, and both groups show elevated risks compared to men. In contrast, for feeling unwell, only women have a statistically significant higher likelihood than men in the full model, while the difference for non-binary respondents is no longer significant. These results suggest that, even after accounting for experiences of violence, non-binary individuals continue to face distinct disadvantages in terms of social exclusion, while gendered patterns of health outcomes are more pronounced for women. The findings reinforce the importance of considering both mediation and intersectionality when analysing workplace inequalities. The findings reinforce the significance of analysing gender beyond binary frameworks and incorporating both mediation and intersectionality in the study of workplace inequalities within Human Resources Management research and policy.

## Concluding discussion

### Contributions to research and theory

The rationale of the study from a Human Resource Management (HRM) perspective is to address the critical challenges of psychological violence and sexual harassment faced by non-binary employees. While a gendered perspective on organisations has become more prominent, the experiences of non-binary people—a minoritised group within a minoritised group—remain largely invisible and unspoken about (Hennekam & Ladge, 2023). Definitions and conflations around the LGBT acronym mask some crucial nuances in relation to experiences of different gender and sexual identity groups at work. Our analysis provides a conceptual differentiation between gender identity, trans status and sexual orientation, as identified and reported by respondents. This provides richer insights about their respective effects on individual experiences. Thus, this study contributes to Rumens (2016, p. 44) call to understand more about the ‘organisational realities’ of non-binary and wider LGBT people and overcome ‘the clumsiness of constituting acronyms such as LGBT in so much as they imply a shared single identity among people who differ considerably.’ The study therefore fills gaps in the HRM literature by providing empirical evidence on the unique experiences of non-binary individuals, particularly within gendered organisational structures that operate on binary gender norms. It adds to the literature on stigma and minority stress beyond the LGB communities, suggesting that non-binary people face distinctive challenges and how being non-binary in cisnormative and binary organisations operates as an invisible stigma (Ragins, 2004).

The study also advances EDI scholarship by demonstrating the mediating role of gender-based violence in the relationship between non-binary identity and adverse workplace outcomes. By situating the experiences of non-binary employees within the framework of minority stress theory, the research highlights that psychological violence and sexual harassment mediate feelings of social exclusion, lack of safety, and poor well-being. These findings extend the application of minority stress theory beyond its traditional focus on LGB individuals, offering a nuanced view of gender identity-related stigma within gendered and cisnormative workplaces. Researching this area from a gender minority stress perspective is important because it sheds light on the unique challenges faced by non-binary individuals in workplaces that are structured around binary gender norms and highlights the systemic and relational dynamics that contribute to the heightened vulnerabilities of non-binary employees. By focusing on non-binary experiences of psychological violence and sexual harassment, it becomes possible to uncover the mechanisms through which cisnormative and binary structures perpetuate harm. Such insights are crucial for designing EDI initiatives that account for the compounded challenges faced by non-binary employees. This underscores the importance of addressing systemic organisational norms and practices that reinforce stigma and expose non-binary individuals to these forms of violence. By integrating gender-based violence into the analysis, the research reframes diversity management as a broader challenge that encompasses both social safety and social equity. As such, this recognises that diversity management is not only about people feeling accepted and included, but creating safe workplaces (Köllen & Rumens, 2022; Ladwig, 2022).

Finally, the article challenges conventional approaches to EDI that may inadvertently uphold binary gender norms and cisnormativity. Our work shows the relevance of temporarily stabilising categories of diversity, within an intersectional analytical framework, for theorising the experiences of non-binary people at work. By foregrounding the experiences of non-binary employees within an intersectional analytical framework, it calls for a re-examination of organisational policies, practices, and cultural norms to disrupt binary thinking and create spaces that are authentically inclusive and safe. Through its empirical and theoretical contributions, the study provides actionable insights for HRM professionals to mitigate minority stress through targeted interventions. These efforts not only support non-binary employees but also foster a culture of genuine diversity, equality, and inclusion that benefits all organisational members.

### Practical implications

HRM practitioners face significant challenges in supporting non-binary employees due to the entrenched binary gender norms that shape organisational policies, practices, and cultures. These norms often result in systemic oversights, such as the lack of gender-inclusive data collection methods, absence of non-binary recognition in legal documents or HR systems, and inadequacies in diversity training. Such structural limitations not only fail to accommodate non-binary identities but also perpetuate feelings of exclusion and marginalisation among non-binary employees, amplifying minority stress. The inability to address these systemic barriers leaves HRM practitioners ill-equipped to foster truly inclusive work environments, further alienating employees whose identities do not align with binary frameworks.

Organisations need to invest in getting better understandings and raise awareness of experiences of employees at work in relation to gender identity, trans status and sexual orientation. There is a need for comprehensive and sophisticated data systems to collect information on such grounds to ensure that diversity management initiatives address different needs and challenges, appreciating the heterogeneity of behaviours, identities and experiences across and within different groups. Without knowledge and evidence, putting in place diversity management policies and practices is challenging (Ozturk & Tatli, 2016). Diversity management can often lead to reproducing gender binarism and cisnormativity within organisations (Bendl et al., 2008). Diversity management efforts therefore need to destabilise and challenge the gender binary status quo (Restar et al., 2021; Tzanakou & Pearce, 2019) to make organisations safe and inclusive for all employees.

For example, various practices have been suggested to create more inclusive workplaces for non-binary employees, such as an open dress code, gender inclusive bathrooms, awareness training, establishing mechanisms to address silencing and facilitate collective voices through staff networks and other activities (Köllen, 2021; Sawyer et al., 2016) or developing safe and brave spaces that could change organisations from within (Ladwig, 2022). However, it is crucial for diversity management efforts to start with an acknowledgement and recognition that organisations are gender binary and cisnormative workplaces (Ladwig, 2022) in order to create the conditions for authentic and honest dialogue about what this entails for those who do not conform to these spaces (‘bodies out of place’) and develop strategies that aim to challenge and change those norms and practices. This means challenging the binary order which permeate data systems, hierarchies, roles, behaviours and scripts around gender and gender identity (Beauregard et al., 2018; Chapman & Gedro, 2009; Pringle, 2008).

But other practices and interventions are possible yet often neglected, because gender-based violence is not sufficiently considered. A key issue for HRM practitioners is addressing the heightened vulnerability of non-binary employees to gender-based violence, including psychological violence and sexual harassment. These experiences are both a cause and a consequence of minority stress, as non-binary individuals are subjected to stigmatisation and victimisation due to their nonconformance to binary gender norms. HRM professionals often lack the tools and frameworks needed to identify and mitigate these risks effectively, particularly in workplaces where reporting mechanisms are underutilised due to fears of retaliation or lack of confidence in organisational responses. This failure to create protective measures can exacerbate the stressors faced by non-binary employees, who may feel unsafe and unsupported at work.

Despite this call for better diversity initiatives, we want to stress the importance of recognising that they may have unintended consequences (Leslie, 2019; Tzanakou & Pearce, 2019). Based on Leslie’s typology, diversity efforts that aim to challenge cisnormativity and gender binaries could make employees feel ‘discomfort’, reduce their engagement with diversity initiatives (‘negative spillover’) and even increase their discriminatory behaviour against the targeted group (in our case the non-binary employees) who might feel fell welcome and thus leave the organisation resulting to reducing representation of the targeted group (‘backfire and negative diversity goal progress’). These negative effects can be mitigated when diversity practices fall within non-discrimination and accountability practices (Leslie, 2019).

Ultimately, a focus on gender-based violence with a view for organisations to become more inclusive is needed. Extending diversity management programmes to include gender-based violence makes the connection more evident as non-binary people are most at risk of psychological violence and sexual harassment. For organisations to become more inclusive, they need to tackle how violence is often a response to those that do not conform to gender norms and the gender binary. This entails organisational commitment and knowledge about the role of gender-based violence in preventing true inclusion at work, that is focusing not only on acceptance and inclusion, but also safety.

### Limitations and future research directions

One limitation of this study lies in its reliance on quantitative methods, which, while providing robust statistical insights, may oversimplify the complexities of non-binary identities and experiences. The use of fixed categories for gender identity does not fully capture the fluidity and diversity of non-binary individuals. This conceptual reductionism could limit the depth of understanding necessary for better diversity management interventions. Incorporating gender fluidity into quantitative measurements of gender diversity in future surveys will prove to be a challenging yet beneficial undertaking. Future research should also integrate qualitative methods, such as interviews or ethnographic approaches, to explore the nuanced realities of non-binary employees and their experiences of gender-based violence within organisational settings.

Another limitation concerns the geographic focus of the study, which is restricted to European higher education institutions. While this context offers valuable insights into systemic issues within a specific sector, the findings may not be fully generalisable to other regions or industries. Cultural, legal, and organisational differences across countries and sectors could result in varying experiences of non-binary employees, as well as different challenges and opportunities for HRM practitioners. Future studies should expand the geographic and sectoral scope to include diverse organisational and cultural contexts, enabling a more comprehensive understanding of non-binary workplace experiences globally.

Finally, the cross-sectional nature of the data constrains the ability to examine changes over time or the long-term impact of interventions aimed at supporting non-binary employees. Without longitudinal data, it is challenging to assess whether organisational efforts to address psychological violence, sexual harassment, and minority stress are effective in creating safer and more inclusive workplaces. Future research should employ longitudinal designs to track the outcomes of diversity management initiatives and their impact on the well-being and career progression of non-binary employees.

## Data Availability

The data that support the findings are available from the Leibniz Institute for the Social Sciences (GESIS) at https://doi.org/10.7802.2475.
